# Vaccination perception and coverage among healthcare students in France in 2019

**DOI:** 10.1186/s12909-020-02426-5

**Published:** 2020-12-14

**Authors:** Aurélie Baldolli, Jocelyn Michon, Renaud Verdon, Anna Fournier

**Affiliations:** 1 Department of Infectious Diseases, CHU de Caen, Avenue de la Côte de Nacre, F-14000 Caen, France; 2 Groupe de Recherche sur l’Adaptation Microbienne (GRAM 2.0), Université Caen Normandie, F-14000 Caen, France

**Keywords:** Vaccination perception, Vaccination hesitancy, Vaccination coverage, Healthcare students

## Abstract

**Background:**

Vaccine hesitancy in healthcare workers has been increasing especially in France while they are the cornerstone of vaccination programs. Greater understanding of healthcare students (HCS) vaccine knowledge, attitudes and beliefs is necessary to provide an adequate vaccination education to better equip them to promote vaccination in their future careers. The aim of this study was to assess vaccination perception (VP) (perception of benefits and risks of vaccines) and its impact on vaccination coverage (VC) for mandatory and recommended vaccines among HCS.

**Methods:**

A standardized, anonymous self-reporting electronic questionnaire was prospectively sent to HCS (medicine, nursing, pharmacy, midwifery, physiotherapy students and 1st year of health sciences students) of Normandy University in France between 18/03/2019 and 8/04/2019. VP was evaluated with questions regarding vaccination hesitancy, safety of vaccine and the benefit/risk balance of vaccination. Global VC (GVC) was defined as being vaccinated according to the mandatory and/or recommended vaccination schedule by national French law in 2018.

**Results:**

542 HCS took part in this survey. VC was high for mandatory (diphtheriae, poliomyelitis, tetanus 93.5%, hepatitis B virus 88.6%) and even most of recommended vaccinations (measles 95%, pertussis 88.2%). Global VC (40.4%) was not statistically different between HCS except for 1st year health sciences students who were less vaccinated (25.6%). Regarding VP, 97.8% of HCS thought that vaccine are effective. When vaccine safety and level of vaccine hesitancy were assessed (on a 0–10 scale, 0: not safe or not hesitant and 10: completely safe and strongly hesitant for vaccine), 91% of respondents stated that vaccine safety is ≥7 and in 80% the vaccine hesitancy was < 3. There was no difference among student categories. 80.6% of HCS recommended all vaccines but only 52% agreed that flu vaccination should be mandatory for HCS. In the multivariate analysis, being a 1st year health care sciences student was associated with a lower GVC (OR 95% CI = 2 [1.2–3.3], *p* = 0.004) than being a medical student.

**Conclusion:**

HCS perceived vaccine as effective and secure. Despite the good perception of vaccines, less than half HCS are well vaccinated.

**Supplementary Information:**

The online version contains supplementary material available at 10.1186/s12909-020-02426-5.

## Background

Vaccination is considered as one of the most successful public health measures of the twentieth century in combating infectious diseases [1, 2]. According to the World Health Organization, 2 to 3 millions of lives are saved each year through vaccination [1]. However for a few decades, vaccination has been perceived as unsafe and unnecessary by a growing number of individuals in the world, especially in most western countries [3–5]. Vaccine hesitancy (VH) is defined by the SAGE Working Group on Vaccine Hesitancy as “the delay in acceptance or refusal of vaccination despite availability of vaccination services” [3]. VH is complex and context specific, varying across time, place and vaccines. It is influenced by factors such as complacency, convenience and confidence [3]. In recent years, a high negative perception of vaccine safety has been observed in France, with an estimated prevalence of VH of 46% among parents of 1 to-15-year old children and 35% among elderly individuals [4, 5]. Despite positive preliminary results of the extension of mandatory infants vaccination against 11 diseases in France in 2018, where mothers were more favorable to mandatory vaccination with an increase vaccination rates, many people in France are still hesitant about vaccination [6]. Recently, an online survey in a representative sample of the French population, found that 26% of respondents would not use a vaccine against SARS-CoV-2 if it became available [7]. The perception of the benefits and risks of vaccines plays a role in the development of vaccine hesitancy. Indeed, several studies show that individuals who perceive vaccines as less necessary and safe, more often reject scheduled vaccinations for their children and for themselves [8–10]. Parental vaccine hesitancy is a growing problem affecting the health of children and a larger population. The main consequence of this VH is insufficient vaccination coverage (VC), leading to an increased risk of outbreaks such as measles outbreaks in France in 2010–11, accounting for more than half of the 30,000 cases in Europe during this period and numerous sporadic outbreaks are still occurring in Europe [11–13]. VH is a real challenge for healthcare workers (HCWs), especially general practitioners (GPs), who are the cornerstone of vaccination programs in many countries. HCWs advice plays an important role in patients’ behavior towards vaccination and are one of the strongest influencers in parent’s vaccination decision. Systematic reviews show that greater trust in HCWs, especially GPs is associated with a higher likelihood of accepting vaccines [8–10, 14]. Although HCWs generally approve of vaccination, several studies have shown doubts among them about the usefulness and safety of vaccines [15–20]. In a recent European study, main concerns of VH of HCWs were the fear of side effects, especially regarding new vaccines, the lack of information and communication about side effect and a strong mistrust of pharmaceutical companies [20]. It is well demonstrated that HCWs with low confidence in the benefits and safety of vaccine will less recommend vaccine to their patients, their children and themselves [17, 20–22]. Two French studies, regarding VH of GPs and their practices regarding vaccination, showed that 16 to 43% of them sometimes or never recommended at least one specific vaccine, VH being in in the south-east of France [17, 23]. Their recommendation behaviors depend on their trust in authorities and information sources, their perception of the utility and risks of vaccines, and their comfort in explaining them to patients [17, 23]. Intervention strategies to restore confidence in vaccine should target GPs and other HCWs. One of the strategy could be to improve vaccine education of health care students (HCS). Indeed findings suggest that there is an important gap in the knowledge of graduating nursing, medical and pharmacy trainees regarding vaccine indications, side effects and safety [24, 25]. Greater understanding of HCS vaccine attitudes and beliefs is necessary to provide an adequate vaccination education to better equip them to promote vaccination in their future careers.

The aim of this study was to assess HCS vaccination perception (VP) (assessed as the perception of benefits and risks of vaccines) and its impact on their own VC for mandatory and recommended vaccinations.

## Methods

### Study design and setting

A self-reporting electronic questionnaire (14 questions) related to VP and VC based on the WHO survey Questions Hesitancy in French provided by the WHO’s Strategic Advisory Group of Experts (SAGE) on Immunization was developed for this study (supplementary data) [26]. The WHO SAGE on immunization have proposed examples of survey questions designed to assess determinants of vaccine hesitancy [26]. We have chosen some questions among them to develop our survey. Between 18/03/2019 and 8/04/2019 the questionnaire was proposed to the second year students of each school of HCSs of Normandy University (2 medical schools, 8 nursing schools, 2 pharmacy schools, 2 midwifery schools, 3 physiotherapy schools), to the 1st year of the Première Année Commune d’Etudes de Santé (PACES) (which is a first year common core for physicians, physiotherapists, midwives, dentists, and pharmacists) and to the 3th to 6th year medical school students (2 schools). On the 18/03/2019 a link to the Limesurvey questionnaire was prospectively sent to them by e mail. It was also available on the students’ association facebook wall for the duration of the study and each week, a reminder email was sent to all students.

### Data collection and definitions

A standardized, anonymous, self-administered questionnaire designed specifically for the study was used to collect information regarding demographic characteristics (sex, age, health study category, and Normandy district of birth (Calvados, Orne, Manche, Eure and Seine-Maritime)), vaccination status for French mandatory (diphtheria, tetanus, poliomyelitis (DTP)) and recommended vaccines (pertussis, hepatitis B virus (HBV), measles, mumps and rubella (MMR), meningococcal C, human papillomavirus (HPV) for women and varicella if not immunized) regarding the French 2019 guidelines for vaccinations [8]. HBV vaccination is mandatory for HCWs but is only recommended for the general population [8]. Data about VC were recorded on the basis of self-report. A tutorial was associated with the questionnaire to help students analyze their own vaccination notebook. Questions evaluated VH, benefit/risk balance of vaccination, safety and efficiency of vaccination, agreement or disagreement with the recent French law requiring infants to be immunized with 11 vaccines within 2 years of life and their opinion with influenza vaccine among health care providers. Global VC was defined as being vaccinated for French mandatory and recommended vaccines according to the French 2019 guidelines for vaccinations [27]. Not being up to date for global VC was defined as not being vaccinated with one of those vaccines. Vaccination hesitancy was evaluated on a 0–10 scale: 0 for no hesitancy regarding vaccination and 10 for maximal hesitancy. Vaccination safety was also evaluated on a 0–10 scale: 0 for vaccination is not safe and 10 for vaccination is safe. VP was defined as the result of the assessment of benefit/risk balance of vaccine in general.

### Statistical analysis

Continuous variables were expressed as medians, and categorical variables were reported as percentages. Univariate analysis of not being up to date with GV was conducted using χ2 and Fisher exact tests. All factors with *p* values lower than 0.2 were integrated into a multivariate logistic regression model. Odds ratios (ORs) and their 95% confidence intervals (CIs) were used as measures of association. A *p* value lower than 0.05 was considered to be statistically significant.

Due to a small number of respondents in some health care categories, we did the univariate and the multivariate analysis with three health care categories (medical, paramedical (nurse, pharmacist, midwifes, physiotherapist) and PACES students). All analyses were performed with STATA v14.0 (StataCorp, College Station, TX, USA). This study was approved by the ethics committee of Caen Hospital (ID 256) and was performed in accordance with the Declaration of Helsinki.

## Results

### Study population characteristics

Out of a population of 4546 HCSs, 542 (11.9%) took part in this survey: 117 were PACES (21.6%), and 284 were medical (52.4%), 31 were pharmacy (5.7%), 86 were nursing (15.9%), 14 were physiotherapy (2.6%) and 10 were midwifery students (1.8%). Their median age was 22.3 years, and 79.0% were female (Table 1). Response rate was 4 times higher for medical students (22.08%) than for nurse or physiotherapist (6.89 and 5.56% respectively).

**Table 1:** Demographic characteristics of all the respondents and by the health care category of health care students

	All respondents *n* = 542	Medical student *n* = 284	Nurse student *n* = 86	Pharmacist student *n* = 31	Physiotherapist student *n* = 14	Midewife student *n* = 10	PACES student *n* = 117
**Sex n(%)**							
Male	113 (20.8)	74 (26)	8 (9.3)	6 (19.4)	3 (21.4)	1 (10)	25 (21.4)
Female	429 (79.2)	210 (74)	82 (90.7)	25 (80.6)	11 (78.6)	9 (90)	92 (78.7)
**District of birth n(%)**							
Calvados	166 (30.6)	96 (33.8)	12 (14)	5 (16.1)	1 (7.1)	2 (20)	50 (42.7)
Manche	103 (19)	62 (21.8)	13 (15.1)	6 (19.3)	1 (7.1)	1 (10)	20 (17.1)
Orne	55 (10.1)	31 (10.9)	6 (7)	2 (6.5)	3 (21.4)	1 (10)	12 (10.3)
Seine Maritime	77(14.2)	29 (10.2)	31 (36)	9 (29)	5 (35.7)	2 (20)	1 (0.9)
Eure	26 (4.8)	8 (2.8)	8 (9.3)	3 (9.7)	3 (21.4)	1 (10)	3 (2.6)
Others	115 (21.2)	58 (20.4)	16 (18.6)	6 (19.4)	1 (7.1)	3 (30)	31 (26.5)
**Years of graduate medical school n(%)**							
2nd year		53 (18.7)					
3th year		54 (19)					
4th year		70 (24.6)					
5th year		54 (19)					
6th year		53 (18.7)					
**Mean age (years)**	22.3	22.2	20.9	23.0	22.2	27.1	20.7

### Vaccination perception

**Fig. 1:**
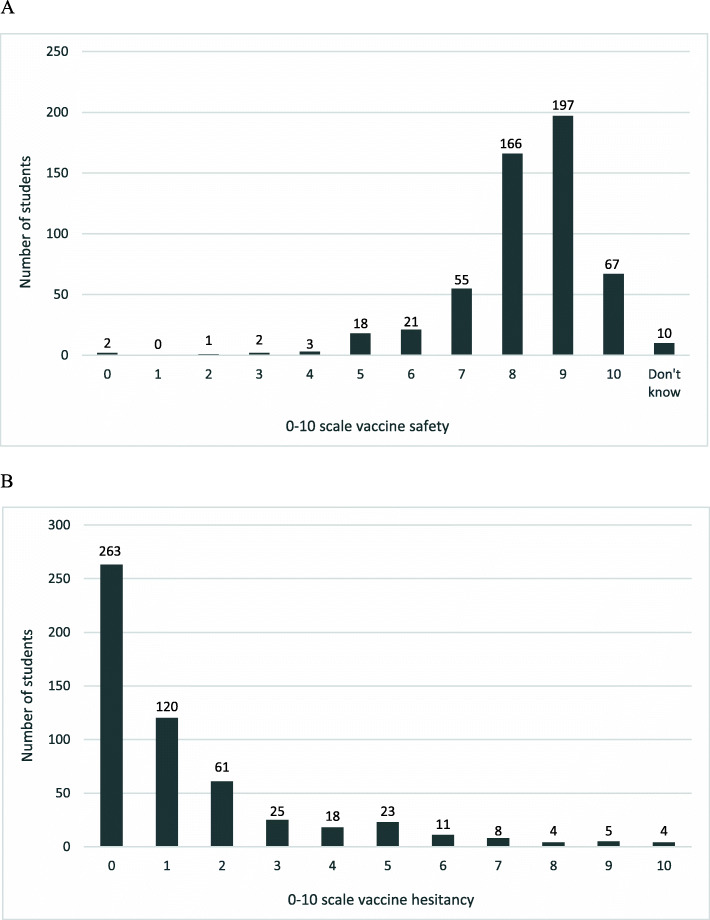
Vaccine safety (**a**) and vaccine hesitancy (**b**) among healthcare students. Vaccine safety (**a**) and vaccine hesitancy (**b**) among healthcare students as measured using a numeric scale. 0 for no hesitancy to 10 for maximal hesitancy; 0 for vaccination not safe to10 for vaccination is safe

Regarding VP, 97.6% of students thought that vaccines were effective. On a 0–10 scale, 91% thought that vaccine safety was ≥7, and 80% had vaccine hesitancy < 3 (Fig. 1a and b). The benefit/risk balance for vaccination was judged as always positive by 399/542 HCSs (73.6%), positive for only some vaccinations by 122/542 HCSs (22.5%) and always negative by 9/542 HCSs (1.6%). In those HCSs who had negative or impartial opinions (*n* = 122), incriminated vaccines were the zona (88/122, 72.1%), HPV (70/122, 57.4%), *Haemophilus influenza* b (Hib) (74/122, 60.7%) and flu (72/122, 59%) vaccines and less frequently the meningococcal C (43/122, 35.2%), MMR (32/122, 26.2%) and DTP (30/122, 24.6%) vaccines. All vaccines were recommended by 80.6% of HCSs (437/542) and some vaccines only were recommended by 15.5% (84/542). For these 15.5% of students who did not recommend all vaccines (*n* = 84), the Hib (58/84, 69%), HPV (53/84, 63.1%), influenza (60/84, 71.4%), zona (69/84, 82.1%) and meningococcal vaccines (39/84, 46.4%) had the highest reluctance rate. There was no difference according to student categories. The recent French law requiring infants to be immunized with 11 vaccines within 2 years of life was approved by 91.5% (496/542). However, only 52.0% (282/542) of HCSs agreed that flu vaccination should be mandatory for HCWs. The proposal of mandatory influenza vaccination was disapproved by 39.8% of medical (113/284), 62.4% of paramedical (88/141), and 50.4% of PACES students (59/117).

### Vaccination coverage

Only 40.5% of students reported having their vaccination notebook when they completed the questionnaire. The characteristics of student VC are reported in Table 2. Vaccination rates were high for mandatory vaccinations (DTP 93.5%) and even for most of the recommended vaccinations (MMR 94.8%, pertussis 88.6%, HBV 88.2%). VC for HPV and meningococcus C was low (*n* = 276, 50.9% and *n* = 335, 61.8%, respectively). GVC (*n* = 219, 40.4%) was not significantly different between medical (*n* = 125, 44%), nursing (*n* = 39, 45.3%), midwifery (*n* = 40, 40%), pharmacy (*n* = 16, 51.6%) and physiotherapy students (*n* = 5, 35.7%). GVC without HPV was higher than GVC with HPV (53.9% versus 40.4%). However, PACES students were less vaccinated (*n* = 30, 25.6% and *n =* 39, 33.3%) than medical students for GVC with and without HPV vaccination ((*n* = 125, 44% and *n* = 165, 58.1% respectively) and especially for the HBV vaccine (61.5% versus 96.5%) and the meningococcus C vaccine (53.8% versus 60.2%). Physiotherapy students were also less vaccinated for pertussis and meningococcus C than other students (PACES and paramedical students) (71.4 and 50%). A past history of varicella was reported in 80% (*n =* 437), and 8.1% (*n* = 44) did not know their varicella immunization status. Among subjects without a history of varicella, only 44 were vaccinated.

**Table 2:** Vaccination coverage among healthcare students

	VC All students *n =* 542	VC physicians *n =* 284	VC nurses *n =* 86	VC physiotherapists *n =* 14	VC pharmacists *n =* 31	VC midwife *n =* 10	VC PACES *n =* 117
DTP^a^ n (%)	507 (93.5)	271 (95.4)	81 (94.2)	13 (92.9)	30 (96.8)	10 (100)	102 (87.2)
Pertussis^b^ n (%)	480 (88.6)	260 (91.5)	77 (89.5)	10 (71.4)	28 (90.3)	8 (80)	95 (81.2)
HBV^c^ (3 doses) n (%)	478 (88.2)	274 (96.5)	82 (95.3)	14 (100)	27 (87.1)	9 (90.0)	72 (61.5)
HPV^b^ n (%)	276 (50.9)	139 (48.9)	52 (60.4)	8 (57.1)	17 (54.8)	5 (50.0)	55 (47)
MMR^b^ (2 doses) n (%)	514 (94.8)	272 (95.8)	81 (94.2)	11 (78.6)	31 (100)	9 (90.0)	109 (93.2)
Meningococcus C^b^ n (%)	335 (61.8)	171 (60.2)	58 (67.4)	7 (50)	26 (83.9)	7 (70.0)	63 (53.8)
Global VC n (%)	219 (40.4)	125 (44.0)	39 (45.3)	5 (35.7)	16 (51.6)	4 (40.0)	30 (25.6)
Global VC without HPV n (%)	292 (53.9)	165 (58.1)	55 (64.0)	6 (42.9)	22 (71.0)	5 (50.0)	39 (33.3)

^a^mandatory

^b^recommended

^c^mandatory in healthcare worker

*DTP* diphteriae, tetanus, poliomyelitis, *HBV* hepatitis B virus, *HPV* human papillomavirus, *MMR* measles, mumps, rubella, *VC* vaccination coverage

### Risk factors for not being up to date with mandatory and recommended vaccinations

#### Univariate analysis

On univariate analysis, age, sex, the district of birth, years of study or belief that vaccines are safe < 6 on a 0–10 scale, or not effective, were not associated with VC. However, being a PACES student was found to be a risk factor for being less vaccinated (OR 2.3 [1.4–3.7] *p* = 0.0008) than medical students, as well as for disagreement with the French mandatory vaccination requirement in infants (OR 1.8 [1.2–2.7] *p* = 0.004) (Table 3).

**Table 3:** Risk factors of not being up-to-date for global vaccination among HCS

	N (%)	Univariate analysis Odd ratio [IC 95%]	***p***	Multivariate analysis Odd ratio [IC 95%]	***p***
**Sex**					
Male	113 (20.8)	–	0.25		
Female	429 (79.2)	1.3 [0.8–1.9]			
**Healthcare category**					
Medical	284 (52.4)	–	0.0008*	–	0.004*
Paramedical	141 (26)	0.9 [0.6–1.4]		0.8 [0.5–1.2]	
PACES student	117 (21.6)	2.3 [1.4–3.7]		2.0 [1.2–3.3]	
**District of birth**					
Calvados	166 (30.6)	–	0.06		
Manche	103 (19)	0.7 [0.5–1.2]			
Orne	55 (10.1)	1.2 [0.6–2.3]			
Seine Maritime	77(14.2)	0.4 [0.3–0.8]		–	
Eure	26 (4.8)	0.9 [0.4–2.0]			
Others	115 (21.2)	0.7 [0.4–1.2]		–	
**Years of graduate medical school**		–	0.77		
2nd year	53 (18.5)	0.6 [0.3–1.3]			
3th year	54 (19)	0.8 [0.4–1.6]			
4th year	70 (25)	0.7 [0.3–1.5]			
5th year	54 (19)	0.7 [0.3–1.5]			
6th year	53 (18.5)				
**Age (years)**					
20–21	290 (53.5)	–	0.09		
22–23	155 (28.6)	0.8 [0.5–1.1]		–	
24–25	57 (10.5)	0.6 [0.3–1.0]		–	
> 25	40 (7.4)	1.4 [0.7–2.9]			
**Vaccine safety**					
No < 6	110 (20.3)	–	0.21		
Yes ≥6	432 (79.7)	0.7 [0.4–1.3]			
**Vaccine efficiency**					
Yes for all vaccines	15 (2.8)	–	0.10		
Yes for some vaccines	514 (94.8)	0.5 [0.2–1.6]			
“I don’t think so”	10 (1.8)	3.3 [0.3–34.7]			
I don’t know	3 (0.6)	0.7 [0.1–10.4]			
**French mandatory vaccination extension on infant**					
Agree	496 (91.5)	–	0.004*	–	0.04*
Disagree	46 (8.5)	1.8 [1.2–2.7]		1.6 [1.1–2.5]	
**Will you recommend vaccination?**					
Yes for all vaccines	246 (56%)	–	0.005*		
Yes for some vaccines	61 (72.6%)	2.1 [1.2–3.4]			
No	16 (76.2%)	2.5 [0.9–6.9]			

*: *p* < 0.05

#### Multivariate analysis

In the multivariate analysis, being a PACES student was associated with a lower GVC (OR 95% CI = 2 [1.2–3.3], *p =* 0.004) than being a medical student. Disagreement with the French mandatory vaccination requirement in infants was also found to be an independent risk factor for not being up to date with vaccinations (OR 95% CI = 1.6[1.1–2.5], *p* = 0.04) (Table 3).

## Discussion

To our knowledge, this is the first study evaluating VP among HCSs and its relationship with VC. Our study showed that HCSs had a good VP, with more than 90% of them thinking that vaccines were effective and safe. However, the belief that vaccines were effective and safe was not significantly associated with GVC, which was low in our study (40.4% for GVC with HPV vaccine and 53.9% without HPV vaccine). The only risk factor associated with low GVC in multivariate analysis were being a PACES student and disagreement with the French mandatory vaccination requirement in infants.

These results could be explained by the fact that HCSs are usually young (less than 25 years old) and healthy. Despite a good VP, they rarely see their general practitioner and vaccination schedules might be impacted by a missed immunization. In two French studies, in general medical practice patients aged 13 to 24 year-old account for 8% only of the patients, and vaccination represent 8.3 and 1.9% of consultations in patients younger than 16 years-old and patients older than 16 years-old, respectively [28, 29]. In French 2019 guidelines for vaccinations, mandatory and recommended vaccines (HPV, MMR, HBV, DTP, Pertussis, Meningococcus C) have to be done during the childhood and the adolescence before starting health care studies [27]. After the PACES year, a systematic check of at least mandatory vaccines for HCS (DTP and HBV) is performed by the preventive medical department or the healthcare university service [27]. This leads to an increased VC against DTP and HBV in other HCS compared to PACES students. Regarding recommended vaccines, the reported VC for MMR in our study (2 doses) was higher (95%) than most of previous French studies [30–34]. Due to measles outbreaks since 2010 in France with more than 10,000 cases, the vaccination recommendations were changed in 2011 from one to two doses for individuals 20–30 years old [12, 13, 35]. Previous French studies have been realized between 2010 and 2013 during the period where measles vaccination changed. This can partially explain that their VC for MMR (2 doses) was low, ranging from 13.6 to 78%, with no difference between nurse, medical or midwife students. However, even for 1 dose MMR, VC rate was lower and varied in most other studies from 62.8 to 85.7%, reflecting the fact that at this period there was probably a lack of knowledge of recommended occupational vaccinations in this population [31, 32, 34, 36]. Our result may have been over estimated with only 40% of HCSs reported using their vaccination notebook to complete the questionnaire. However MMR vaccine acceptance is high in France with more than 75% of acceptance among hospital HCWs and GPS recommended MMR vaccine in more than 80% of cases (despite a suboptimal VC for MMR at the same period) [23, 31].

Surprisingly, VC for HPV in HCS was high (64%) compared to 20-year-old girls in France (22.18%) in 2018 [13]. HPV vaccine is only recommended in France for girls from 11 to 19 years old or homosexual men up to 25 years old [27]. In 2013, only 72.4% of GPS recommend always or often HPV vaccine for girls [23]. VC for HPV is relatively low in France while GP seems to be confident with this vaccine. However, numerous studies have demonstrated that medical professionals often failed to communicate effectively about this vaccine with patients and parents, especially if they felt uncomfortable discussing sex, perceived parents as hesitant, or believed patients to be at low risk [37]. There was no significant difference between HCS categories in our study for VC for HPV. No data are available regarding HPV VC among new HCW or HCS. However, our results probably reflect that HCSs are more convinced of the utility of immunization for HPV.

VP in our study was good, with no difference regarding healthcare category. VP did not impact VC among HCS in multivariate analysis. This can be explained by the fact that most of HCS had a good perception (safety, efficiency, benefit/risk) of vaccine. Although VP was good, only 52% of HCSs agreed that flu vaccination should be mandatory for HCWs. Many studies have found that influenza VC remains low in HCSs, especially in paramedical staff, due to a fear of side effects, the idea of low efficacy of this vaccine and the lack of awareness of the importance of herd immunity [15, 16, 38, 39]. In this study, while they have a better medical knowledge, 39.8% of medical students did not approve mandatory flu vaccine in HCWs. Unfortunately, we did not evaluate the reason for a negative response on the questionnaire. Few studies also reported a low influenza VC among physicians and an increased vaccination hesitancy among general practitioners [16, 34, 36, 40, 41]. Nevertheless, medical students, had a better immunization rate than for other HCSs, probably because they are more informed regarding the spread of infectious diseases and more receptive to immunizations. It is difficult to draw strong conclusions for some HCS categories which are underrepresented, such as midwifes or physiotherapy students.

As a consequence of a low VC among HCSs, nosocomial cases of vaccine-preventable disease have been reported, such as for influenza or even measles and pertussis [19, 20]. New strategies to increase VC among HCSs, especially for PACES students, should be discussed. To define such a strategy, studies should search for a correlation between, VC, VP and vaccination knowledge. Some authors have studied knowledge, attitudes and beliefs among HCS towards vaccination [25, 42, 43]. They found a significant variability between immunization education among health care categories. In Dysband ‘s work, high knowledge score of immunization were achieved by 74.3% of medical students, 62.7% of pharmacy students and only 57.1% of nursing students with a higher negative attitudes toward vaccination. Participants in all the three programs (medical, pharmacy and nurse) showed a lack of confidence in addressing the risk of vaccine [25]. In the study of Ghandory, which evaluated the knowledge, attitudes and beliefs of HCS regarding mandatory influenza vaccination, only 51 to 64.5% of student supported the mandatory policy vaccination. Pharmacist students had a higher mean knowledge score than medical and nursing students [42].

It is important that future health care providers have a good knowledge of vaccine (indication, risks) but also being confident in their ability to initiate and guide the vaccine conversations with patients and parents. A large assessment of immunization education in Canadian Health Professional Programs was performed with a survey of 77 questions distributed to all Canadian medical, pharmacy and nursing school. They showed that the time spent to immunization education varied from less than one hour to more than 50 h. In this study, 74% of respondents felt not comfortable in answering parents or patients questions about side effects and only 21.2% felt they received an adequate teaching about vaccines [24]. In France, immunization education varies from medical, pharmacist, midwife and nursing schools and represents less than 10 h of lessons. These results showed the need to improve educational interventions and lectures dedicated to vaccination.

Our work showed that immunization education should be provided as soon as the first year of health science education or the first year of nursing training. Educational program should include different parts. Firstly, teaching lessons about vaccines, types of vaccines, how they works, their immunogenicity, efficacy, efficiency and side effects. These lessons should be repeated during their studies because vaccine programs evolve during time. Then, a vaccine group training should present different practical situations that future HCW and physicians could face in their future careers. For example, a training could about “how to convince parents who don’t want their child to be vaccinated”, would help students to gain more insights in managing vaccinal hesitancy. Moreover, the influenza outbreak period should be used to reassure HCSs of the utility of immunization. This would require full cooperation between the hospital preventive department and the healthcare university service. Students could help these departments during the influenza vaccine campaign at the hospital for health care providers to make them aware of this vaccine.

This study has some limitations. First, the response rate of this survey was low (11.9%) which may create a response bias and limit its generalizability. We tried to improve the response rate with a reminder email every week during the 3 weeks period of the study without effect. We don’t have a good explanation for this low response. Health care students use to work at the hospital for their educational trainee and they may not have consulted their university email. However, it still represent a large population compared to other studies about this topic where, despite a higher response rate, varying from 23.7 to 26%, the numbers of HCS included ranged from 223 to 250, which iss significantly lower than in our study [25, 34]. The second limit of this work is that the data on vaccination status were self-reported, and only 40% of HCSs reported using their vaccination notebook to complete the questionnaire. Results of VC could be over or underestimated. We tried to limit this bias with a tutorial which was associated with the questionnaire to help students analyze their own vaccination notebook. Of note, the average vaccination rate for DTP vaccines in HCSs in France varies from 95.9 to 96.8% in different studies, which is consistent with the average rate in our study [32–34]. Third, the questionnaire that we used was developed for this study and has not been validated in other samples. However, the validity of this survey was assessed by experts (3 infectious diseases doctors, 1 virologist) in the field and the questionnaire was developed based on the WHO survey Questions Hesitancy provided by the WHO’s Strategic Advisory Group of Experts (SAGE) on Immunization, which is already validated in studies related to VH or VP.

Fourth, because of the low response rate of this survey some health care categories are underrepresented such as midwives, physiotherapists or pharmacists. Therefore, results should be interpreted with caution. To limit this bias in the analysis of the impact of VP on VC we performed the univariate and multivariate analysis by pooling students in 3 groups, medical students, paramedical students and PACES students.

## Conclusions

HCSs perceived vaccines as effective and safe. Despite a good perception of vaccines, GVC was low, especially regarding recommended vaccines, and less than half of HCSs were well vaccinated. VP did not seem to impact VC in this study. Some vaccines were considered unuseful or not indicated. Information regarding these vaccines should be provided with a focus on PACES students. Finally, our results suggest that vaccine education should start early in health care studies.

## Supplementary Information

**Additional file 1.** Supplementary data.

## Data Availability

The datasets used and/or analyzed during the current study are available from the corresponding author on reasonable request.

## Abbreviations

DTP: Diphtheria, tetanus, poliomyelitis
CIs: Confidence intervals
GPs: General practitioners
HCS: Healthcare students
HCW: Healthcare workers
HBV: Hepatitis B virus
HPV: Human papillomavirus
MMR: Measles, mumps and rubella
ORs: Odds ratios
PACES: 1st year of health sciences students
VC: Vaccination coverage
VH: Vaccine hesitancy
VP: Vaccination perception
