# Supplementary material for: Vaccination perception and coverage among healthcare students in France in 2019
Source: BMC Med Educ. 2020 Dec 14;20:508. doi: 10.1186/s12909-020-02426-5 (PMC7734903; doi:10.1186/s12909-020-02426-5)
Supplement: Supplementary file 1 — Additional file 1. Supplementary data. [file 12909_2020_2426_MOESM1_ESM.docx]

**QUESTIONNAIRE (translated from French)**

**Demographic information:**

1. **Sex**:  Male  Female
2. **Healthcare category**:  Medical  Nurse  Pharmacist  Physiotherapist  Midwife PACES student
3. **District of birth** :  Calvados  Manche  Orne  Seine-Maritime  Eure  Others…….
4. **Years of graduate medical school:** 2nd year - 3th year - 4th year - 5th year - 6th year
5. **Age (years):** ………

**Vaccination perception :**

1. **Do you think that the following vaccines (listed below) are effective ?**

 Yes for all vaccines

Yes for some vaccines:

Diphtheria, tetanus, poliomyelitis

Measles, mumps and rubella

Hepatitis B

Pneumococcal pneumonia

 Meningococcus invasive infection

Human papillomavirus (condylomas, cervical cancer)

Influenza

*Haemophilus influenza b* meningitis

Pertussis

 Shingles

 I don’t know

 I don’t think so

 No

1. **In general, how do you evaluate vaccine safety on a 0-10 scale?** …………………

**(0 for “vaccination is not safe” and 10 for “vaccination is safe”)**

1. **What do you think about the benefit/risk balance of vaccination ?**

 Positive for all vaccines

 Positive for some vaccines

 Negative for all vaccines

1. **How do you evaluate your own vaccine hesitancy on a 0-10 scale?** …………………

**(0 for “no hesitancy regarding vaccination” and 10 for “maximal hesitancy”)**

1. **Would you recommend vaccination?**

 Yes for all vaccines

Yes for some vaccines:

Diphtheria, tetanus, poliomyelitis

Measles, mumps and rubella

Hepatitis B

Pneumococcal pneumoniae

 Meningococcal invasive infection

Human papillomavirus (condylomas, cervical cancer)

Influenza

*Haemophilus influenza b* meningitis

Pertussis

Shingles

 No

1. **What do you think about the French mandatory vaccination extension (11 vaccines) in all infants within 2 years of life ?**

Agree

Disagree

1. **Do you think that influenza vaccination should be mandatory for health care workers ?**

Yes

No

**Vaccination Coverage :**

1. **Are you up to date with mandatory and recommended vaccinations that are listed below ?**

| Diphtheria, tetanus, poliomyelitis |  yes (5 to 6 doses)  no   don’t know |
| --- | --- |
| Hepatitis B |  yes (3 doses)  no   don’t know |
| Pertussis |  yes (4 to 5 doses)  no   don’t know |
| Measles, mumps and rubella |  yes ( 2 doses)  no   don’t know |
| Human papillomavirus |  yes (2 to 3 doses)  incomplete (1 dose)   no   don’t know   not applicable (male) |
| Meningococcal C |  yes (1 dose)  no   don’t know |
| Varicella |  not indicated : varicella during childhood   yes ( 2 doses)  no   don’t know |

You can go to this link to have the French guidelines for vaccinations to help to complete this question.

<https://solidarites-sante.gouv.fr/IMG/pdf/calendrier_vaccinal_mars_2019.pdf>

1. **Did you have your vaccination notebook when you completed this questionnaire :**

 Yes  No
